# The impact of symptoms on daily life as perceived by patients with Charcot-Marie-Tooth type 1A disease

**DOI:** 10.1007/s10072-021-05254-7

**Published:** 2021-04-26

**Authors:** Stefano Tozza, Dario Bruzzese, Daniele Severi, Emanuele Spina, Rosa Iodice, Lucia Ruggiero, Raffaele Dubbioso, Aniello Iovino, Francesco Aruta, Maria Nolano, Lucio Santoro, Fiore Manganelli

**Affiliations:** 1 Department of Neuroscience, Reproductive and Odontostomatological Science, University of Naples “Federico II”, Via Sergio Pansini, 5, 80131 Naples, Italy; 2 Department of Public Health, University of Naples “Federico II”, Naples, Italy

**Keywords:** CMT1A, Hereditary neuropathy, Ranking approach, Daily life, Quality of life

## Abstract

**Introduction:**

In Charcot-Marie-Tooth type 1A (CMT1A) patients, daily life is mainly influenced by mobility and ambulation dysfunctions. The aim of our work was to evaluate the perception of disturbances that mostly impact on daily life in CMT1A patients and its difference on the basis of age, gender, disability, and quality of life.

**Methods:**

Forty-one CMT1A patients underwent neurological assessment focused on establishing clinical disability through the Charcot-Marie-Tooth Neuropathy Score (CMTNS) and quality of life through the Short Form-36 (SF-36) questionnaire. We identified from CMT disturbances 5 categories [weakness in lower limbs (WLL), weakness in upper limbs (WUL), skeletal deformities (SD), sensory symptoms (SS), balance (B)] and patients classified the categories from the highest to the lowest impact on daily life (1: highest; 5: lowest). Ranking of the 5 categories, in the overall sample and in the different subgroups (dividing by gender, median of age and disease duration, CMTNS, domains of SF-36), was obtained and differences among subgroups were assessed using a bootstrap approach.

**Results:**

Rank analysis showed that WLL was the most important disturbance on daily life whereas WUL had the lowest impact. In the older CMT1A group, the most important disturbance on daily life was B that was also the most relevant disturbance in patients with a greater disability. SD influenced daily life in younger patients. SS had less impact on daily life, with the exception of patients with a milder disability.

**Discussion:**

Our findings demonstrated that the perception of disturbances that mostly impact on CMT1A patients’ daily life changes over the lifetime and with degree of disability.

## Introduction

Charcot-Marie-Tooth disease (CMT) is a hereditary motor sensory neuropathy and Charcot-Marie-Tooth type 1A (CMT1A), a demyelinating form due to duplication of *PMP22* gene, represents the most frequent subtype [1]. CMT patients usually develop symptoms during childhood or adolescence with pes cavus, progressive muscular atrophy and weakness of distal segments of lower and then upper limbs, sensory loss, and decrease or absence of deep tendon reflexes [2].

Disability is related to progressive axonal loss [3–5] and accumulates slowly over time with acceleration of clinical disability after 50 years of age [6]. Several symptoms influence quality of life (QoL) such as mobility, balance, distal motor and sensory limitations, fatigue, pain, and body image [7]. However, the symptoms that most impact on QoL are the ability to ambulate independently and toe-heel walk [8].

The aim of our work was to evaluate the impact of disturbances as perceived by CMT1A on daily life: by using a rank approach, we aimed to understand if all disturbances have the same impact on daily life and if they changed on the basis of age, gender, disability, and QoL.

## Methods

CMT1A patients were recruited and evaluated in a tertiary care neuromuscular center at the University of Naples “Federico II” (Naples, Italy). Inclusion criteria were genetic confirmation of *PMP22* gene duplication and age ≥ 18 years. All patients gave informed consent to participate in the study, which was approved by the local ethics committee of the University of Naples “Federico II” (Naples, Italy).

We assessed clinical disability through the second version of the Charcot-Marie-Tooth Neuropathy Score (CMTNS), a composite scale that includes symptoms, signs, and neurophysiology, amounting to a total range of 0–36 [9].

We assessed QoL through the Short Form-36 (SF-36) questionnaire, a self-reported measure of health, that comprises 36 questions which cover two dimensions (SF-36_Physical and SF-36_Mental) [10].

Moreover, based on disturbances typically referred by CMT patients, we grouped them in five categories:

- weakness in lower limbs (WLL);
- weakness in upper limbs (WUL);
- skeletal deformities (SD);
- sensory symptoms (SS);
- balance (B).

We asked patients to classify the disturbance categories from the highest to the lowest impact on daily life (1: highest impact; 5: lowest impact). Patients reported the disturbance category when it was not complained.

### Statistical analysis

Standard descriptive statistics were used to describe the characteristics of the sample (median with range, mean ± standard deviation, and frequencies with percentages).

Ranking of the 5 disturbance categories, in the overall sample and in the different subgroups (dividing by gender, median of age and disease duration, CMTNS, domains of SF-36), was obtained using the branch-and-bound algorithm as already defined [11]. Differences among subgroups were assessed using a bootstrap approach. For each classification factor, the Kendall’s tau distance between the two rankings was computed. Then, 999 bootstrap samples were drawn without replacement, in order to preserve the original distribution of the classification factor. In each bootstrap sample, the Kendall’s tau distance was computed. *p*-values were finally obtained as the proportion of bootstrap replicates with a Kendall’s tau statistics lower than or equal to that computed on then original sample. In case of multiple solutions in the branch-and-bound algorithm, the pair with the largest distance was chosen. All the analyses were performed using the statistical platform R (vers. 3.5.2) and *p*-values less than 0.05 were deemed as statistically significant.

## Results

Forty-one CMT1A patients were recruited (14 males and 27 females; 50.3 ± 17.8 years, ranging from 22 to 81 years of age). Demographic and clinical information was summarized in Table 1.

**Table 1:** Demographic and clinical data

Male/female	14/27					
Age	50.3 ± 17.8 (22–81)					
CMTNS	15.2 ± 7.1 (1–29)					
SF-36_Mental	58.5 ± 22.2 (8.8–96.7)					
SF-36_Physical	55 ± 22.3 (7.8–93.3)					
Prevalence of ranking positions for each disturbances in the overall cohort						
	1	2	3	4	5	Not complained
Weakness in lower limbs	36.6%	22%	24.4%	12.2%	2.4%	2.4%
Balance	24.4%	19.5%	22%	14.6%	14.6%	4.9%
Skeletal deformities	14.6%	26.8%	19.5%	24.4%	9.8%	4.9%
Sensory symptoms	17.1%	14.6%	17.1%	17.1%	29.3%	4.9%
Weakness in upper limbs	4.9%	17.1%	12.2%	26.8%	31.7%	7.3%

Overall, the category with the highest impact on daily life in our CMT1A group was WLL (mean ranking 2.26). It was followed by B and SD (mean ranking 2.79), SS (mean ranking 3.44), and WUL (mean ranking 3.71) (Table 2).

**Table 2:** Overall consensus ranking

	Consensus ranking	Mean ranking
Weakness of lower limbs	1	2.26
Balance	2	2.79
Skeletal deformities	2	2.79
Sensory symptoms	4	3.44
Weakness of upper limbs	5	3.71

**Fig. 1:**
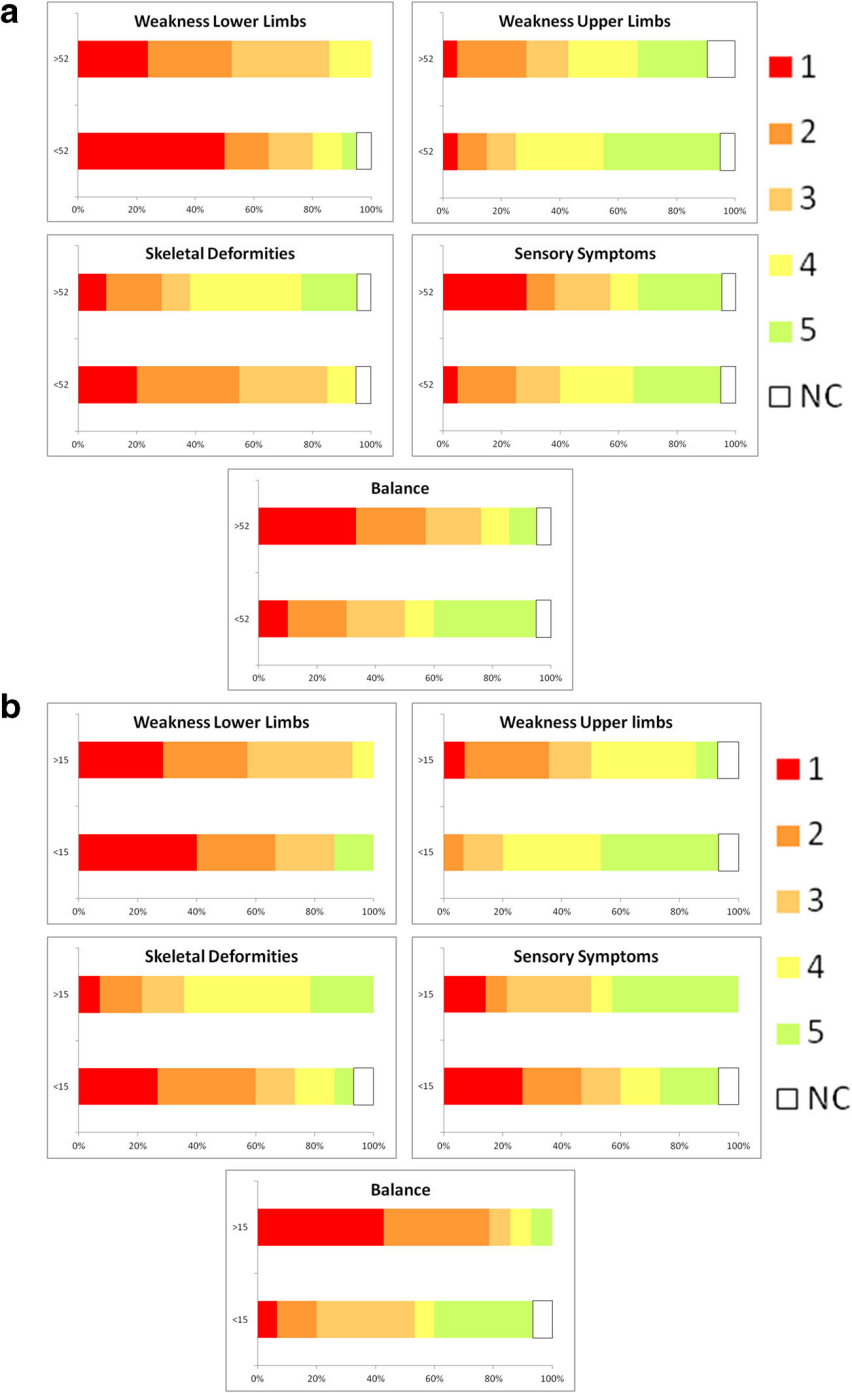
Distribution of disturbance categories. For each disturbance categories, the rank score distribution (1: highest impact; 5: lowest impact; NC: not complained) was reported dividing by median age (**a**) and CMTNS (**b**)

The consensus ranking analysis, dividing by the median age of 52 years of age, showed significant change (*p* = 0.013) of rank between the two group: the youngest CMT1A patients referred WLL as the category with the highest impact on daily life, whereas B represented the category with the highest impact on daily life in elderly CMT1A patients (Table 3; Fig. 1a). Similar results was obtained dividing by the median years of disease duration (20 years): the CMT1A patients with shorter disease duration referred WLL as the category with the highest impact on daily life, whereas B represented the first category with highest impact in CMT1A patient group with a longer disease duration (*p* = 0.011) (Table 3).

**Table 3:** Consensus ranking divided by different variables

	Consensus ranking		
Gender	Male	Female	*p*-value
Weakness of lower limbs	1	2	0.621
Skeletal deformities	2	3	
Sensory symptoms	4	4	
Weakness of upper limbs	5	5	
Balance	3	1	
Age (years)	Age ≤ 52	Age > 52	
Weakness of lower limbs	1	2	**0.013**
Skeletal deformities	2	5	
Sensory symptoms	4	4	
Weakness of upper limbs	5	3	
Balance	3	1	
Disease duration (years)	Duration ≤ 20	Duration > 20	
Weakness of lower limbs	1	2	**0.011**
Skeletal deformities	2	4	
Sensory symptoms	3	5	
Weakness of upper limbs	5	3	
Balance	4	1	
CMTNS	CMTNS ≤ 15	CMTNS > 15	
Weakness of lower limbs	1	2	**0.006**
Skeletal deformities	3	4	
Sensory symptoms	2	5	
Weakness of upper limbs	5	3	
Balance	4	1	
Mental domain SF-36	Mental ≤ 54.4	Mental > 54.4	
Weakness of lower limbs	1	1	0.352
Skeletal deformities	3	2	
Sensory symptoms	4	5	
Weakness of upper limbs	5	4	
Balance	2	3	
Physical domain SF-36	Physical ≤ 57.7	Mental > 57.7	
Weakness of lower limbs	1	1	0.943
Skeletal deformities	3	2	
Sensory symptoms	4	4	
Weakness of upper limbs	5	5	
Balance	2	3	

In bold are reported significant *p*-value (*p* < 0.05)

Dividing by the median score of CMTNS of 15, the analysis showed significant change (*p* = 0.006) of rank between the two groups: the most severe CMT1A patients referred B as the category with the highest impact on daily life, while the patients with mild phenotype complained SS, after WLL (Table 3; Fig. 1b).

The consensus ranking analysis, dividing by the gender, the median value of SF-36_Physical (≤57.7), and SF-36_Mental (≤54.4), showed changes in ranking between groups but they were not significant (*p* > 0.05) (Table 3).

## Discussion

This study showed that patients perceive weakness in lower limbs as the most important disturbance that impacts on their daily life [8] whereas weakness in upper limbs has the lowest impact in CMT1A patients. This is not surprising since weakness typically occurs, at the beginning, in distal lower limb muscles and subsequently spreads to the upper extremities [12]. Confirming this, weakness in upper limbs passed through the last position of rank in younger group to the third position in the elderly CMT1A group.

Instead, imbalance is complained as first disturbance in the elderly CMT1A group (>52 years), in patients with longer disease duration (>20 years), and in patients with a greater disability as assessed by CMTNS (>15). Balance disorder in patients with CMT1A appears to be related to the weakness of both anterior and posterior leg muscles rather than proprioception involvement [13, 14]. During the course of CMT1A lifetime, posterior muscles’ involvement adds to the anterior muscles’ weakness resulting in a greater disability (worsening of the sub item “strength legs” of CMTNS) and in an increased postural instability [15]. Intriguing, CMT1A patients perceive as balance disorders what the clinician considers as a motor involvement.

Interestingly, skeletal deformities (e.g., pes cavus) impact on daily life especially in younger CMT1A patients. Indeed, pes cavus is the second disturbance reported by younger patients while it seems to be scarcely influencing daily life in the older patient group. This suggests that younger patients may pay a greater attention to esthetic that should not be overlooked by clinicians in managing CMT1A patients. Moreover, it would be also possible that young patients pay more attention to skeletal deformities because they want and need to walk and perform better than older patients. On the other hand, elderly patients neglect skeletal deformities since they consider most relevant other disturbances (e.g., balance).

Overall, sensory symptoms have less impact on daily life in CMT1A patients. Nevertheless, sensory symptoms are the second most important disturbance in the group with milder disability (CMTNS ≤ 15). Motor items are more representative of increased disability [16, 17] and therefore it is conceivable that, in CMT1A patients with higher CMTNS, sensory symptoms may be covered by motor disturbances.

Importantly, the disturbance categories were not related to the SF-36 findings suggesting that, regardless what disturbance is in first position, CMT1A patients perceive a low QoL. This data corroborated the “disability paradox” found in CMT population [6, 18] This paradox explains the discrepancy between the QoL and disability that do not correlate in CMT patients.

In conclusion, although these findings should be confirmed through a multicenter study, we demonstrated that the perception of disturbances that impact on daily life changes over the lifetime and with degree of disability. This is relevant for clinicians for a proper approach to CMT1A patients.

## Data Availability

Not applicable.

## Code availability

NEUS-D-20-02411.

## Abbreviations

B: balance
CMT1A: Charcot-Marie-Tooth type 1A
CMTNS: Charcot-Marie-Tooth Neuropathy Score
QoL: quality of life
SF-36: Short Form-36
SD: skeletal deformities
SS: sensory symptoms
WLL: weakness in lower limbs
WUL: weakness in upper limbs
